# Influence of Care Pathway on Thyroid Nodule Surgery Relevance: A Historical Cohort Study

**DOI:** 10.3390/jcm9072271

**Published:** 2020-07-17

**Authors:** Solène Castellnou, Jean-Christophe Lifante, Stéphanie Polazzi, Léa Pascal, Françoise Borson-Chazot, Antoine Duclos

**Affiliations:** 1 Endocrinology Department, Groupement Hospitalier Est, Hospices Civils de Lyon, 69500 Bron, France; francoise.borson-chazot@chu-lyon.fr; 2 Endocrine Surgery Department, Centre Hospitalier Lyon Sud, Hospices Civils de Lyon, 69310 Pierre Bénite, France; jean-christophe.lifante@chu-lyon.fr; 3 Health Services and Performance Research Lab (EA 7425 HESPER), Université Claude Bernard Lyon 1, 69100 Villeurbanne, France; stephanie.polazzi@chu-lyon.fr (S.P.); antoine.duclos@chu-lyon.fr (A.D.); 4 Health Data Department, Hospices Civils de Lyon, 69003 Lyon, France; lea.pascal@chu-lyon.fr

**Keywords:** thyroid nodule, care pathway, guidelines, fine-needle aspiration cytology, thyroid cancer

## Abstract

Background: Guidelines recommend using fine-needle aspiration cytology (FNAC) to guide thyroid nodule surgical indication. However, the extent to which these guidelines are followed remains unclear. This study aimed to analyze the quality of the preoperative care pathway and to evaluate whether compliance with the recommended care pathway influenced the relevance of surgical indications. Methods: Nationwide historical cohort study based on data from a sample (1/97th) of French health insurance beneficiaries. Evaluation of the care pathway of adult patients operated on between 2012 and 2015 during the year preceding thyroid nodule surgery. The pathway containing only FNAC was called “FNAC”, the pathway including an endocrinology consultation (ENDO) with FNAC was called “FNAC+ENDO”, whereas the no FNAC pathway was called “NO FNAC”. The main outcome was the malignant nature of the nodule. Results: Among the 1080 patients included in the study, “FNAC+ENDO” was found in 197 (18.2%), “FNAC” in 207 (19.2%), and “NO FNAC” in 676 (62.6%) patients. Cancer diagnosis was recorded in 72 (36.5%) “FNAC+ENDO” patients and 66 (31.9%) “FNAC” patients, against 119 (17.6%) “NO FNAC” patients. As compared to “NO FNAC”, the “FNAC+ENDO” care pathway was associated with thyroid cancer diagnosis (OR 2.67, 1.88–3.81), as was “FNAC” (OR 2.09, 1.46–2.98). Surgeries performed in university hospitals were also associated with thyroid cancer diagnosis (OR 1.61, 1.19–2.17). Increasing the year for surgery was associated with optimal care pathway (2015 vs. 2012, OR 1.52, 1.06–2.18). Conclusions: The recommended care pathway was associated with more relevant surgical indications. While clinical guidelines were insufficiently followed, compliance improved over the years.

## 1. Introduction

The high clinical prevalence of thyroid nodules reaches 5.3–6.4% in women and 0.8–1.3% in men in countries in which iodine intake is sufficient. The prevalence of palpable thyroid nodules increases with age, reaching more than 20% in women after 50 years of age [1,2,3]. However, their ultrasound (US) prevalence is much higher, reaching more than 60%, and the widespread use of imaging has led to an increase of fortuitous thyroid nodule discoveries over the last years [2,4]. The primary aim of a thyroid nodule investigation is to rule out a malignant tumor. Guidelines recommend performing a Thyroid Stimulating Hormone (TSH) assay for every nodule, as well as a cervical US [5,6,7,8,9,10]. Depending on both the size and the features of the nodule, fine-needle aspiration cytology (FNAC) can be required. Cytology results obtained by FNAC determine the need for surgery but are not an automatic indication for surgery [8,11,12,13]. Surgical indications are standardized and must be restricted to suspicion of malignant, toxic, or compressive nodule. The remaining types of nodules, which are by far more frequent, may be only monitored instead of surgery [14], with active surveillance even being proposed in some instances for papillary thyroid microcarcinoma, a histological type of cancer with low evolutionary potential and very good prognosis [15,16,17]. Indeed, the increased detection of thyroid nodules has led to an increase in the number of thyroid surgeries, resulting in an overtreatment of thyroid nodules and an excess of avoidable surgeries. Such surgeries can lead to hypothyroidism and complications, such as hypoparathyroidism or recurrent laryngeal nerve paralysis, which are responsible for quality of life deterioration and increased health expenditure [18,19,20,21]. The increase in the number of surgeries coupled to the improvement in histology techniques has led to an increase in thyroid cancer incidence over the past 30 years, while thyroid cancer mortality remains low and stable. This fits the definition of overdiagnosis, which involves approximately 70% to 80% of thyroid cancers in France [16,22,23,24,25,26,27,28]. Guidelines should allow for better targeting of surgical indications, leading to fewer avoidable surgeries and fewer fortuitous microcancer discoveries. This would have a beneficial effect on patient quality of life, which can be impaired following thyroid surgery or when a thyroid cancer is discovered [29,30]. However, compliance with those guidelines and factors affecting this compliance are difficult to assess. Studies conducted in France in 2010 and in Germany in 2006 found low rates of FNAC prior to thyroid surgery [1,31], while surveys performed in several countries found a heterogeneous management of thyroid nodules [32,33,34]. Moreover, if some studies suggest that FNAC is the most selective examination for preoperative cancer diagnosis [1,35,36,37], the impact of this examination on surgery relevance in real practice still needs to be studied.

We hypothesize that better compliance with clinical guidelines and the recommended care pathway for thyroid nodule management is associated with improved adequate surgical indications and a decrease in thyroid surgeries performed for benign nodules. The aims of this study were to assess guideline compliance by studying the quality of the preoperative care pathway, to evaluate whether compliance with the recommended care pathway influences the relevance of surgical indications, and to identify the determinants of compliance with the recommended care pathway.

## 2. Materials and Methods

### 2.1. Study Design and Data Source

A historical cohort study was conducted on patients who underwent a surgical procedure for thyroid nodules. Their exposure to care pathways during the 12 months preceding the thyroid surgery was evaluated. Three different types of care pathways were studied. Care pathways were considered optimal when at least a FNAC was performed in accordance with guidelines and non-optimal when no FNAC was performed. In “FNAC+ENDO (endocrinology consultation)” and “FNAC” care pathways a FNAC was performed, however unlike “FNAC”, the “FNAC+ENDO” care pathway included an additional consultation with an endocrinologist. “NO FNAC” care pathway was defined by the absence of FNAC.

To retrace the care pathway of in- and out-patients, data were obtained from a general sample of French health insurance beneficiaries (*Echantillon Général des Bénéficiaires*, EGB). The EGB is a permanent representative sample based on a survey at the 97th percentile on the health insurance number of the French nationwide health data system (*Système National des Données de Santé*, SNDS). The SNDS is an exhaustive and anonymous data warehouse that gathers sociodemographic, care reimbursement, and care pathway information regarding both hospital and ambulatory care for the entire French population. Reimbursement information includes medical or paramedical procedures and consultations delivered, as well as medications, presence of a long-term disease, biological sampling, and radiological examination. Those data are matched with hospital care information that pertains International Classification of Diseases 10 (ICD-10) diagnoses codes and surgical procedures performed in all public and private hospitals.

### 2.2. Study Population

Adult patients who had a thyroid nodule surgery, including both single nodule and multinodular thyroids (included based on the ICD-10 codes C73, D09.3, D34, D44.0, E01.1, E04.1, and E04.2), between 1 January 2012, and 31 December 2015, in metropolitan France were included in the study. Herein, the index hospital stay refers to the stay during which the thyroid surgery was performed. Patients operated for hyperthyroidism, goiter other than multinodular, or with concurrent parathyroid resection were excluded from the study. In order to consider only patients without medical history of thyroid cancer, patients whose index surgical procedure was a thyroid completion were excluded. Furthermore, when considering the 12 months preceding the index hospital stay, patients in long-term disease treatment for thyroid cancer or who had a thyroid surgery, a cervical lymph node dissection, a radioactive iodine treatment, or a diagnosis of thyroid cancer reported during a hospitalization were also excluded from the study.

### 2.3. Outcome

The main outcome measure was the nature of the nodule on histopathological analysis. Nodules were considered malignant if a diagnosis of thyroid cancer (ICD-10 codes C73 and D09.3) was recorded during the index hospital stay. Nodules initially considered as benign, whose nature was not specified or uncertain at discharge, were secondarily reclassified as malignant if patients received a radioactive iodine treatment, entered the care pathway for long-term diseases specific to thyroid cancer, were operated for a thyroid completion or for a lymph node dissection, or if a diagnosis of thyroid cancer was recorded during a secondary hospitalization in the 12 months following the surgery date. Patients without cancer diagnosis were divided into 2 categories depending on the ICD-10 codes reported during the index hospital stay: benign nodules (D34, D44.0, E04.1) and multinodular goiter (E01.1, E04.2).

### 2.4. Statistical Analysis

Continuous variables were reported as the median and range (minimum and maximum), while categorical variables were reported as counts and percentages. For the bivariate analysis, continuous variables were compared between patients with thyroid cancer and the others using Wilcoxon rank-sum test, while categorical variables were compared using Chi-squared test. To evaluate the association between the type of care pathway (“FNAC+ENDO”, “FNAC”, “NO FNAC”) and the probability of having a thyroid cancer, and to identify factors associated with an optimal care pathway, stepwise multivariate logistic regression models were performed, with thyroid cancer (yes vs. no), and optimal care pathway (“FNAC+ENDO” or “FNAC” vs. “NO FNAC”) as the dependent variables, respectively. Patient (age, gender, socioeconomic status) and hospital (university hospital or non-university hospital) characteristics, patient area of residency (Paris area or other), and the year of the surgery were considered as covariates. Socio economic status was obtained using data from the Universal Healthcare Coverage (*Couverture Maladie Universelle*, CMU), and we considered being a beneficiary as a marker of precariousness. Indeed, all persons residing in France are covered by the French health insurance system, which includes people who have been living in France on a stable and regular basis for more than 3 months, whose income is below a certain ceiling, and who are entitled to free care through the “Couverture maladie universelle”. Restricted cubic splines with 3 knots were used to model the effect of age as a continuous covariate. Explanatory variables were included in the multivariate models at *p* < 0.20 (entry level) and exited by the stepwise procedure at *p* ≥ 0.10 (removal level). Two-way interactions were then tested between all variables of the final models with a stepwise procedure (entry level at *p* < 0.20 and removal level at *p* ≥ 0.05). Adjusted odds ratios (OR) were presented with their 95% confidence intervals (95% CI). A *p*-value of less than 0.05 was considered to indicate statistical significance. Data manipulation and analyses were performed using SAS software (version 9.4; SAS Institute Inc., Cary, NC, USA).

### 2.5. Ethical Considerations

This study was strictly observational and based on anonymous data. Therefore, in accordance with the French legislation in place at the time of the study, it did not require the written informed consent of participants or the authorization from an ethics committee. The university hospital of Lyon (*Hospices Civils de Lyon*, HCL), as a health research institute, was authorized to use the EGB database by the National Data Protection Commission (*Commission Nationale de l’Informatique et des Libertés*, CNIL), provided that the researcher followed specific training with certification and recorded their study into the register of EGB studies performed in the institute.

## 3. Results

**Figure 1:**
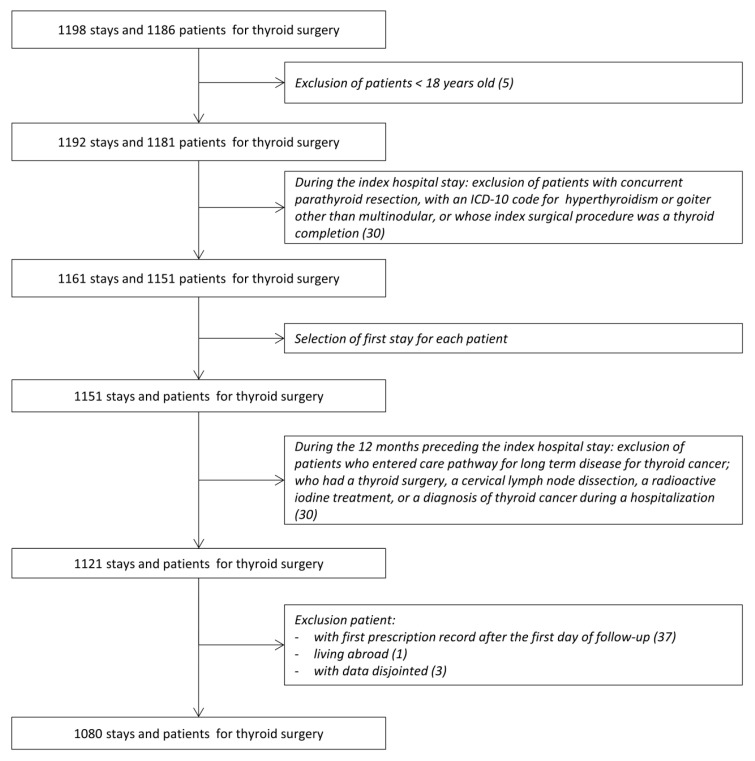
Flow chart.

A total of 1080 patients who underwent a thyroid nodule surgery between 2012 and 2015 were included in the study (Figure 1).

**Figure 2:**
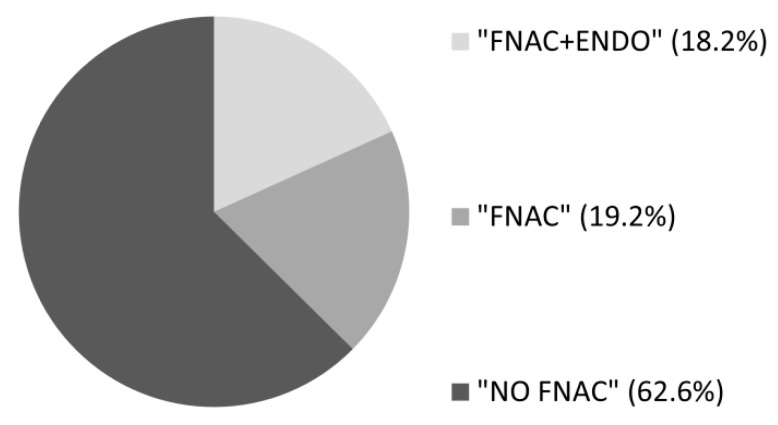
Distribution of the 1080 patients operated on for thyroid nodules between 2012 and 2015 according to the 3 care pathway types. FNAC: Fine-Needle Aspiration Cytology; ENDO: endocrinology consultation.

The majority (77.8%) of patients were females and the median age was 52 years. Overall, a TSH assay was performed in 986 (91.3%) patients, a thyroid US in 906 (83.9%) patients, a FNAC in 404 (37.4%) patients, and a consultation with an endocrinologist in 512 (47.4%) patients. Regarding the type of care pathway, an optimal care pathway was found in 404 (37.4%) patients, with 197 (18.2%) within the “FNAC+ENDO” and 207 (19.2%) within the “FNAC” care pathways, while a non-optimal “NO FNAC” care pathway was found in 676 (62.6%) patients (Figure 2).

Surgery was performed in a university hospital for 326 (30.2%) patients. Multinodular goiter was the main reported diagnosis, found in 487 (45.1%) patients, followed by benign nodule in 336 (31.1%) patients, while 257 (23.8%) operated patients were diagnosed with thyroid cancer (Table 1). 

In the bivariate analysis, factors associated with a diagnosis of cancer were the type of care pathway and the surgery being performed in a university hospital (both *p*-value < 0.001). A cancer diagnosis was recorded in 138 (34.2%) patients of the optimal care pathway, including 72 (36.5%) among patients in the “FNAC+ENDO” pathway, and 66 (31.9%) among patients in the “FNAC” pathway, compared with 119 (17.6%) cancer diagnoses for patients in the “NO FNAC” care pathway. In university hospitals, 100 (30.7%) surgeries were associated with a cancer diagnosis, while the same was true for 157 (20.8%) surgeries performed in non-university hospitals (Table 2).

**Figure 3:**
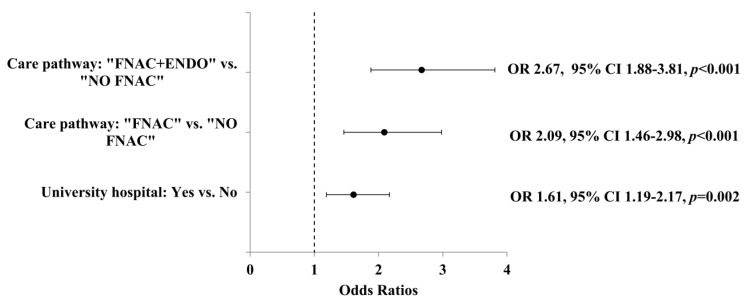
Multivariate analysis of factors having an influence on thyroid cancer diagnosis among patients operated on for thyroid nodules between 2012 and 2015. FNAC: Fine-Needle Aspiration Cytology; ENDO: endocrinology consultation.

These associations remained significant in the multivariate analysis. The association of care pathway type with thyroid cancer diagnosis was stronger for “FNAC+ENDO” (OR 2.67; 95% Confidence interval (CI) 1.88–3.81; *p*-value < 0.001) and “FNAC” (OR 2.09; 95% CI 1.46–2.98; *p*-value < 0.001) than for “NO FNAC”, but the “FNAC+ENDO” care pathway was not significantly more associated with thyroid cancer diagnosis than “FNAC” (OR 1.28; 95% CI 0.85–1.94; *p*-value = 0.243). Surgeries occurring in university hospitals were significantly associated with thyroid cancer diagnosis (OR 1.61; 95% CI 1.19–2.17; *p*-value = 0.002; Figure 3) and patients operated on in a university hospital were more susceptible to benefits from the optimal care pathway, with 141 (43.3%) following the recommended care pathway versus 263 (34.9%) in non-university hospitals (OR 1.39; 95% CI 1.06-1.83, *p*-value = 0.016).

**Figure 4:**
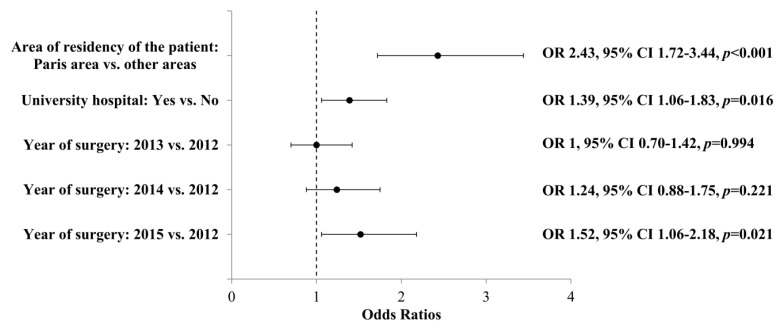
Multivariate analysis of factors having an influence on optimal care pathway compliance among patients operated for thyroid nodule between 2012 and 2015. FNAC: Fine-Needle Aspiration Cytology; ENDO: endocrinology consultation.

Compliance with the recommended care pathway was also associated with patient area of residency, with the likelihood of having an optimal care pathway being higher in the Paris area: 86 (55.8%) patients living in the Paris area had the recommended care pathway versus 318 (34.3%) in other areas (OR 2.43; 95% CI 1.72–3.44, *p*-value < 0.001). The increase in years for surgery tended to be associated with the optimal care pathway; this association was significant in 2015 vs. 2012 (OR 1.52; 95% CI 1.06–2.18; *p*-value = 0.021; Figure 4).

## 4. Discussion

The present population-based study allowed the evaluation in a real-life setting of compliance with guidelines and the association with the relevance of surgical indications for thyroid nodules. Although this study used French data, it is likely that these findings could be generalized, based on the heterogeneity of thyroid nodule management observed in other countries [31,32,33,34,38,39,40,41]. The majority of surgeries were performed for benign pathology, as approximately only one-quarter were associated with a thyroid cancer diagnosis, suggesting an overtreatment of thyroid nodules. Compliance with guidelines was insufficient, since only one-third of patients in the cohort had a FNAC prior to their thyroid surgery. Herein, compliance with the recommended care pathway was associated with a two-fold increase in the likelihood of performing a surgery for thyroid cancer compared to the non-optimal care pathway. Evaluation of the situation and requirement for surgery might be improved if an endocrinology consultation is proposed, as the “FNAC+ENDO” care pathway was found to be more associated with cancer diagnosis than “FNAC”, although not significantly.

The quality and safety of care are at the heart of current priorities. It is mandatory to reduce the number of non-necessary surgeries, can lead to hypothyroidism, and therefore the need for life-long thyroid hormone supplementation, and which are responsible for complications. All this reduces the quality of life of patients and results in medical costs [42]. The discovery of an incidental thyroid cancer, even one with an excellent prognosis such as micropapillary thyroid cancer, can be stressful for the patient, who will require life-long follow-up. In addition, it can have financial consequences for the patient, as a loan may be more difficult to obtain after a cancer diagnosis. FNAC is recommended in all guidelines [7,8,9,10,43] to help select patients requiring surgery, since studies have reported FNAC to be the most useful tool for preoperative diagnosis of thyroid cancer [1,35,36,37,38]. One of the advantages of the present study is that it allowed the evaluation of guideline compliance and the resulting outcome in real practice, in a nationwide historical cohort study, and on patients managed in or outside hospitals. The results herein confirm that guideline compliance improves surgical indication relevance; however, guideline observance is far from systematic. Regional disparities have previously been observed [1] and seem to persist, as highlighted by the fact that the Paris area was more associated with an optimal care pathway than other areas. This could be partly explained by the high medical density and better access to continuous medical education of the Paris area [44]. Surgeries performed in university hospitals were associated with better compliance with the optimal care pathway, potentially highlighting the increased thyroid cancer diagnosis associated with these hospitals. It is, however, important to bear in mind that university hospitals also have a higher recruitment of cancer patients and might be better trained for selection of surgical indication. Differences in thyroid nodule management are also probably due to an inadequate dissemination of information. Guidelines need to be communicated to all actors involved in thyroid pathologies. Various health professionals, such as surgeons or general practitioners, practicing either outside or inside hospitals are involved and need to be trained in order to improve the quality of care pathways and to homogenize thyroid nodule management within the country.

We chose to conduct this study using the EGB, a representative sample of the French population covered by the national health insurance, obtained from the SNDS. Data about medical resource utilization and both hospital and ambulatory care are prospectively collected in an exhaustive way into this rich database, giving access to real-world data. However, although data from the SNDS give access to reimbursement information, the results of examinations, clinical data, as well as the medical history of patients are not precisely available. Since FNAC results were not known, it was not possible to assess whether the surgical indication when a FNAC was performed was truly based on the obtained cytological result. We chose to include patients with multinodular goiter in the study, as multiple thyroid nodules are quite frequent and because suspicious nodules in multinodular goiter are not an uncommon cause for surgery. However, for a certain proportion of patients, surgical indication could have been retained due to symptomatic or progressive goiter. This is probably the case for a certain number of patients in the “NO FNAC” care pathway, as the proportion of patients with multinodular goiter was more important in this group. This indicates that not all surgeries for benign disease are avoidable. Furthermore, a certain proportion of thyroid cancer in the cohort might have been related to fortuitous discoveries, although this is difficult to assess, since symptoms and pathology analysis were unknown [45,46]. A previous study showed that incidental cancer discovery was more common in multinodular goiter than in thyroid operations for a nodule [35]. Therefore, we can assume that the proportion of fortuitous cancer findings was higher in the non-optimal care pathway groups, with the proportion of goiters being higher in this group. Less than one-fifth of patients had both a FNAC and an endocrinology consultation. This could be explained by the fact that in some cases, such as patients undergoing surgery for progressive nodules after several years of follow-up, a FNAC might have been performed initially but would not be repeated before surgery. This could also explain the proportion of patients who did not have FNAC prior to the operation even though they were under the care of an endocrinologist.

FNAC must be performed according to well defined indications, when the size and characteristics of the nodule upon US examination require it [10,13]. All care pathways with FNAC were considered optimal because the size and features of the nodule were not known. It has been reported that more than half of FNAC are unnecessary [47]. Therefore, US needs to be performed by trained radiologists with thyroid expertise to improve pre-operative assessment of thyroid nodules and better assess the need for FNAC, as unnecessary FNAC can lead to diagnosis difficulties and avoidable surgeries. Moreover, FNAC has a high sensitivity for thyroid cancer diagnosis, at around 94–97%, but a poor specificity and positive predictive value, with both estimated to be approximately 50% [13,48]. This could partly explain the low thyroid cancer prevalence (30 to 40%) often observed among operated patients who have had a FNAC prior to surgery [13,36,48,49,50], a result concordant with the result observed herein. This low prevalence also likely reflects the heterogeneity of patients undergoing surgery, as some will be operated on for other reasons than a suspicious malignant nodule. Moreover, cytological analysis needs to be performed by an expert pathologist and the addition of molecular analysis in some cases could also help improve accuracy during optimal care pathway diagnosis. Results should be discussed and coordination between general practitioners, specialists, hospital care, and ambulatory care should be developed in order to help decision-making and enhance surgical indication relevance. The management of thyroid nodules of indeterminate risk of malignancy needs to be more standardized. When possible, and after taking into account the preferences of informed patients, more conservative approaches than surgery should be encouraged. Patient willingness to be operated on or not is indeed an important factor to consider. Informing patients about their pathology and reassuring them in cases of benign nodules is essential to promote shared decision-making and is probably one of the key factors to decrease the number of thyroid surgeries.

Even if there is room for progress, the present results found an improvement in optimal care pathway compliance over time, suggestive of an increase in guideline dissemination. Further studies are needed to understand all factors affecting guideline knowledge and compliance and to establish useful actions to improve these.

## 5. Conclusions

The proportion of thyroid surgery performed for benign nodules is excessively high. This highlights the need to improve risk stratification for better surgical indications. The care pathway recommended in current guidelines is effective in decreasing avoidable surgeries but is insufficiently followed, although improvements have been observed over the years. Interventions are necessary to improve current practice and address geographical disparities.

## Figures and Tables

**Table 1:** Characteristics of the 1080 patients who underwent thyroid nodule surgery between 2012 and 2015 according to their distribution in the 3 care pathways.

	Type of Care Pathway before Surgery			Total
	FNAC+ENDO	FNAC	NO FNAC	
Total	197	207	676	1080
Age (years), median (range)	51.0 (18.0–81.0)	51.0 (18.0–85.0)	53.0 (19.0–84.0)	52.0 (18.0–85.0)
Gender				
Male	42 (21.3)	57 (27.5)	141 (20.9)	240 (22.2)
Female	155 (78.7)	150 (72.5)	535 (79.1)	840 (77.8)
Universal healthcare coverage beneficiaries				
Yes	6 (3.0)	13 (6.3)	29 (4.3)	48 (4.4)
No	191 (97)	194 (93.7)	647 (95.7)	1032 (95.6)
Year of surgery				
2012	53 (26.9)	53 (25.6)	202 (29.9)	308 (28.5)
2013	43 (21.8)	47 (22.7)	175 (25.9)	265 (24.5)
2014	53 (26.9)	55 (26.6)	171 (25.3)	279 (25.8)
2015	48 (24.4)	52 (25.1)	128 (18.9)	228 (21.1)
Patient area of residency				
Paris area	41 (20.8)	45 (21.7)	68 (10.1)	154 (14.3)
Other areas	156 (79.2)	162 (78.3)	608 (89.9)	926 (85.7)
Hospital status				
University hospital	61 (31.0)	80 (38.6)	185 (27.4)	326 (30.2)
Non-university hospital public or private	136 (69)	127 (61.4)	491 (72.6)	754 (69.8)
Examinations performed the year before surgery				
TSH assay	188 (95.4)	179 (86.5)	619 (91.6)	986 (91.3)
Cervical US	175 (88.8)	176 (85.0)	555 (82.1)	906 (83.9)
FNAC	197 (100.0)	207 (100.0)	0 (0.0)	404 (37.4)
Endocrinology consultation	197 (100.0)	0 (0.0)	315 (46.6)	512 (47.4)
Type of index thyroid surgery				
Partial	58 (29.4)	88 (42.5)	181 (26.8)	327 (30.3)
Subtotal or total	139 (70.1)	119 (57.5)	495 (73.2)	753 (69.7)
Complementary surgery at 1 year				
Thyroid completion	7 (3.6)	4 (1.9)	12 (1.8)	23 (2.1)
Lymph node dissection (index stay included)	32 (16.2)	26 (12.6)	28 (4.1)	86 (8.0)
**Outcomes**				
Thyroid pathology				
Cancer	72 (36.5)	66 (31.9)	119 (17.6)	257 (23.8)
Multinodular goiter	66 (33.5)	66 (31.9)	355 (52.5)	487 (45.1)
Benign nodule	59 (29.9)	75 (36.2)	202 (29.9)	336 (31.1)

Data reported as counts (percentages in columns), unless otherwise specified. FNAC: Fine-Needle Aspiration Cytology; ENDO: endocrinology consultation.

**Table 2:** Bivariate analysis of factors susceptible to influence thyroid cancer diagnosis among patients operated on for thyroid nodules between 2012 and 2015.

	Malignancy of the Nodule		Total	*p*-value *
	No	Yes		
Total	823	257	1080	
Type of care pathway				<0.001
FNAC+ENDO	125 (63.5)	72 (36.5)	197	
FNAC	141 (68.1)	66 (31.9)	207	
NO FNAC	557 (82.4)	119 (17.6)	676	
Age (years), median (range)	53.0 (19.0–85.0)	51.0 (18.0–84.0)	52.0 (18.0–85.0)	0.139
Gender				0.175
Male	175 (72.9)	65 (27.1)	240	
Female	648 (77.1)	192 (22.9)	840	
Universal healthcare coverage beneficiaries				0.884
No	786 (76.2)	246 (23.8)	1032	
Yes	37 (77.1)	11 (22.9)	48	
Year of surgery				0.397
2012	237 (76.9)	71 (23.1)	308	
2013	205 (77.4)	60 (22.6)	265	
2014	217 (77.8)	62 (22.2)	279	
2015	164 (71.9)	64 (28.1)	228	
Area of residency of the patient				0.737
Paris area	704 (76.0)	222 (24.0)	926	
Other areas	119 (77.3)	35 (22.7)	154	
Hospital status				<0.001
Non-university hospital, public or private	597 (79.2)	157 (20.8)	754	
University hospital	226 (69.3)	100 (30.7)	326	

Data reported as counts (percentages in lines) unless otherwise specified. * *p*-values from the Wilcoxon rank-sum test for continuous variables and Chi-squared test for categorical variables. FNAC: Fine-Needle Aspiration Cytology; ENDO: endocrinology consultation.
